# Reducing Harm, Supporting Recovery: a partnership and evidence-informed approach to developing the new Irish health led, National Drug Strategy

**DOI:** 10.1186/s12954-019-0348-9

**Published:** 2020-01-08

**Authors:** Catherine Comiskey

**Affiliations:** 1 The National Implementation Committee of the National Drug Strategy, Dublin, Ireland; 2 Professor in Healthcare Statistics, School of Nursing and Midwifery, Trinity College Dublin, The University of Dublin, 24 D’Olier St, Dublin 2, Ireland

**Keywords:** Partnership, Philosophy, Policy, Ireland, Health, Recovery

## Abstract

**Background:**

Policy development by partnership is difficult, however, ‘Reducing Harm, Supporting Recovery- A health led response to drug and alcohol use in Ireland 2017-2025’ hailed a new era. This policy was based on an agreed philosophy and core values across a 21-member partnership and has stated a common commitment to a health-led response.

**Methods:**

To drive strategy development, a cross-discipline committee with an independent Chair was created by the Minister. Members came from statutory, voluntary, community, research and service-user organisations. A consensus-based, partnership approach was taken to developing the policy and the action plan. Over 18 months of debate, a public consultation, focus groups, evidence reviews and an external expert review were conducted. Evidence was reviewed by the committee and following a very robust debate, a set of priority actions and responsible organisations were established.

**Results:**

Nineteen meetings were held. Epidemiological indicators illustrated that cannabis use, young people, chronic opiate use, mortality and geography were a priority. Almost 3000 individuals/organisations responded to the public consultation and themes arising were, supply-reduction, prevention, treatment, rehabilitation and research. The evidence review found that evidence was weak or lacking. The focus groups addressed priorities in supply, education, prevention, continuum of care, evidence and best practice. Finally, the expert review examined structures. Significant contentious debate arose around the initial terms of reference and the authority of a member to agree to an action on behalf of a ministry. While not all members were fully satisfied with the strategy, all welcomed the commitment to the health-led approach. An implementation committee was established, a tender for the first medically supervised injecting facility was issued and a sub-committee to explore decriminalisation was formed.

**Conclusion:**

A key recommendation from the process was to ensure that all voices had an equal opportunity to be heard and to ensure that priority actions identified from the wider sources of evidence were not lost during the extended process. The breath of the partnership aided this. While we have succeeded in developing a sound strategy, success will depend on continuing support from the partnership and appropriate resourcing from the ministries.

## Background

The European Monitoring Centre for Drugs and Drug Addiction (EMCDDA) is responsible for monitoring the drug situation in the EU member states plus Turkey and Norway. In their recent paper on developments in national drug strategies, they highlighted a gradual change in national drug strategies, with new strategies emerging with a broader focus covering other substances and to a lesser extent other addictions [12]. The EMCDDA believes this broader focus will bring not only new opportunities for public health-orientated policies but also new challenges in terms of resources and actions.

Within Ireland, ‘Reducing Harm, Supporting Recovery- A health led response to drug and alcohol use in Ireland 2017-2025’ was hailed as a new era in Irish drug policy [7]. This new policy built on advances made in two previous strategies. The first being, ‘Building on experience: National Drugs Strategy 2001-2008’ [11] and the second, ‘National Drug Strategy (interim) 2009-2016 [6]. These previous strategies focused on reducing harms with a focus on supply reduction, prevention, treatment, rehabilitation and research. They introduced the new era in Irish drug policy, an era where the harm reduction philosophy was clearly stated and aspired to. Ireland as a nation had progressed its vision and underlying philosophy from the abstinence-based, detoxification mentality of the 1980s through the 1990s with the introduction of needle exchange services and increasingly widely available substitution treatment, to a philosophy in the 2000s which recognised the need to strive towards removing stigma, improving well-being and ensuring the basic human rights of all of its citizens [2].

These changes in national political philosophical underpinnings can be traced back to early challenges in the then current philosophies and policies of the 1980s. This is evident in the original 1980s treatment outcome studies which reported that in a follow-up study in 1984 of 88 people who used heroin in 1982, 74 (84%) participants were tracked, and of these, 69 (93%) had attempted to give up heroin, primarily through detoxification programmes [4, 5]. However, in the follow-up study, the role of methadone as a substitution treatment emerged in the findings. It was reported that at the time of the second interview, 11 (15%) of participants were attending methadone programmes and 18 (24%) were heroin free [5]. While a drug-free status may have been the aspiration in the 1980s predominantly catholic Ireland, the wider role and responsibilities of parents, education, church and state were emerging as can be seen in the following:

…attention should be given to: encouragement of social life and of hobbies and sports; improvement in length of education and end achievement; provision of jobs; and discouragement of cigarette smoking and of frequent drinking.…Here then is a challenge: for parents, for the clergy, for teachers, for social workers, health educators, publicans, and government – and, not least, for the young people themselves. ([3], p. 11)*.*

The need for a consensus-based partnership approach to economic and social policy development in Ireland was first introduced in 1987 and was reflected in the sentiments expressed by the Prime Minister who called for greater social inclusion and a new focus on equality ([10], p2).

The aim of this paper is to present the Irish policy development process and to show how three decades after the work of Dean [3], a wide, partnership-led, evidence-informed approach to a health-led policy was developed. The objectives are to present a summary of the evidence base for the policy, describe the partnership and its background and provide examples of how conflicts were addressed and consensus obtained. While this may have been hailed as a new era in Irish drug policy based on an agreed philosophy and core values across a wide partnership, it reflected our roots and most interestingly the challenge of Dean [3] as the partnership had wide representation.

## Methods

To drive the strategy development, the Minister of State with responsibility for drug strategy formed a cross-discipline committee with 21 official members and an independent chair. The Minister invited members from all relevant statutory, voluntary, community, research, and service user organisations. These included senior decision-making civil servant members from each ministry, relevant senior members or chair persons from agencies or networks working with people who use drugs and finally senior independent researchers. Each organisation was invited to propose a relevant representative who had the authority to speak on behalf of the organisation. When the representative could not attend due to a diary commitment, a reserve representative was sent in their place. Positions within organisations and ministries were selected rather than individuals. All organisations who were invited accepted. Some further specific organisations were not invited to the main strategy committee but were invited as part of specific working groups. An example of one such group was the International Nurses Society for Addictions. Members were not remunerated for their time but rather were released from their place of normal work to attend and prepare for the meetings. The committee was fully supported by the Department of Health’s Drug Policy Unit (DPU). A full list of the partnership committee is provided in Table 1.

**Table 1:** Organisational membership of the steering committee of the national drug strategy, Ireland

Sector	Body	Number of representatives
Statutory sector	Department of Health	3
Statutory sector	Health Service Executive	2
Statutory sector	Department of Justice & Equality	1
Statutory sector	An Garda Síochána	1
Statutory sector	Department of Education & Skills	1
Statutory sector	Department of Housing, Planning and Local Government	1
Statutory sector	Department of Children & Youth Affairs	1
Statutory sector	Department of Social Protection	1
Statutory sector	Health Research Board	1
Community sector	Community Sector – represented by CityWide Drugs Crisis Campaign	2
Community sector	National Family Support Network	1
Voluntary sector	Voluntary Sector – represented by the Voluntary Drug Treatment Network	2
Cross-sector task force network	Local Drug and Alcohol Task Force Chairs Network	1
Cross-sector task force network	Regional Drug and Alcohol Task Force Chairs Network	1
Cross-sector committee	National Advisory Committee on Drugs and Alcohol	1
Representative group	UISCE	1

A set of concise terms of reference were developed and agreed following wide ranging debate. Over 18 months, a public consultation, focused or working groups, evidence reviews and an external expert review were conducted. The working groups and the public consultation enabled the partnership to access the views of additional organisations and the general public. National data from the EMCDDA’s five key epidemiological indicators [14] were presented to the committee. The committee met 19 times from late 2015 to mid-2017. Each meeting was of at least 2–3 h duration and members travelled to attend from all over the Republic of Ireland.

All evidence resulting from the various methods was reviewed by the committee. Sections of draft strategy was prepared by the drugs policy unit. These were presented to the full committee and following strong and often difficult debate, independently chaired, sections of strategy were rewritten, debated upon again and finally agreed often by consensus rather than by unanimity. This difficult but transparent process was repeated over the 19 meetings and a final set of priority actions and responsible organisations was established. Some key findings, tensions and resolutions in the process are provided in the following.

## Results

National data from the EMCDDA’s five key epidemiological indicators [14] on general population prevalence of drug use, high risk drug use, treatment demand, drug-related deaths and drug-related infectious diseases were presented to the committee. From these five indicators, it was clear that cannabis use, young people, chronic opiate use, mortality and geography were a priority [13, 15–17].

The Department of Health opened a public consultation on the development of the proposed new national drug strategy. Almost 3000 individuals/organisation responded to the public consultation and overarching themes arising were supply-reduction, prevention, treatment, rehabilitation and research. Further details on the consultation methods and results are available [8].

To allow for a wider group who would ultimately be responsible for delivering the services of the strategy to contribute in key areas, four focused working groups were established and chaired by the chair of the main steering committee. Each member of the particular group was asked to prepare their responses to a set of questions in advance. The time in the group was then used to obtain consensus and a single position paper identifying priorities and practical recommendations was prepared by each group. This paper then went to the main committee for consideration and ultimately for inclusion in the final action plan. Questions posed were the relevance of the current strategy, the identification of gaps from their organisational or professional perspective and an indication of how they thought this gap could be addressed. The four working groups covered the topics of supply reduction, education and prevention, continuum of care and finally evidence and best practice. Full details of the position papers are available from Drugs Policy Unit [9]. All recommendations presented within the group discussion papers were reviewed by the main committee, duplications were noted and removed and finally recommendations were framed as actions and mapped into the action plan of the strategy.

**Fig. 1:**
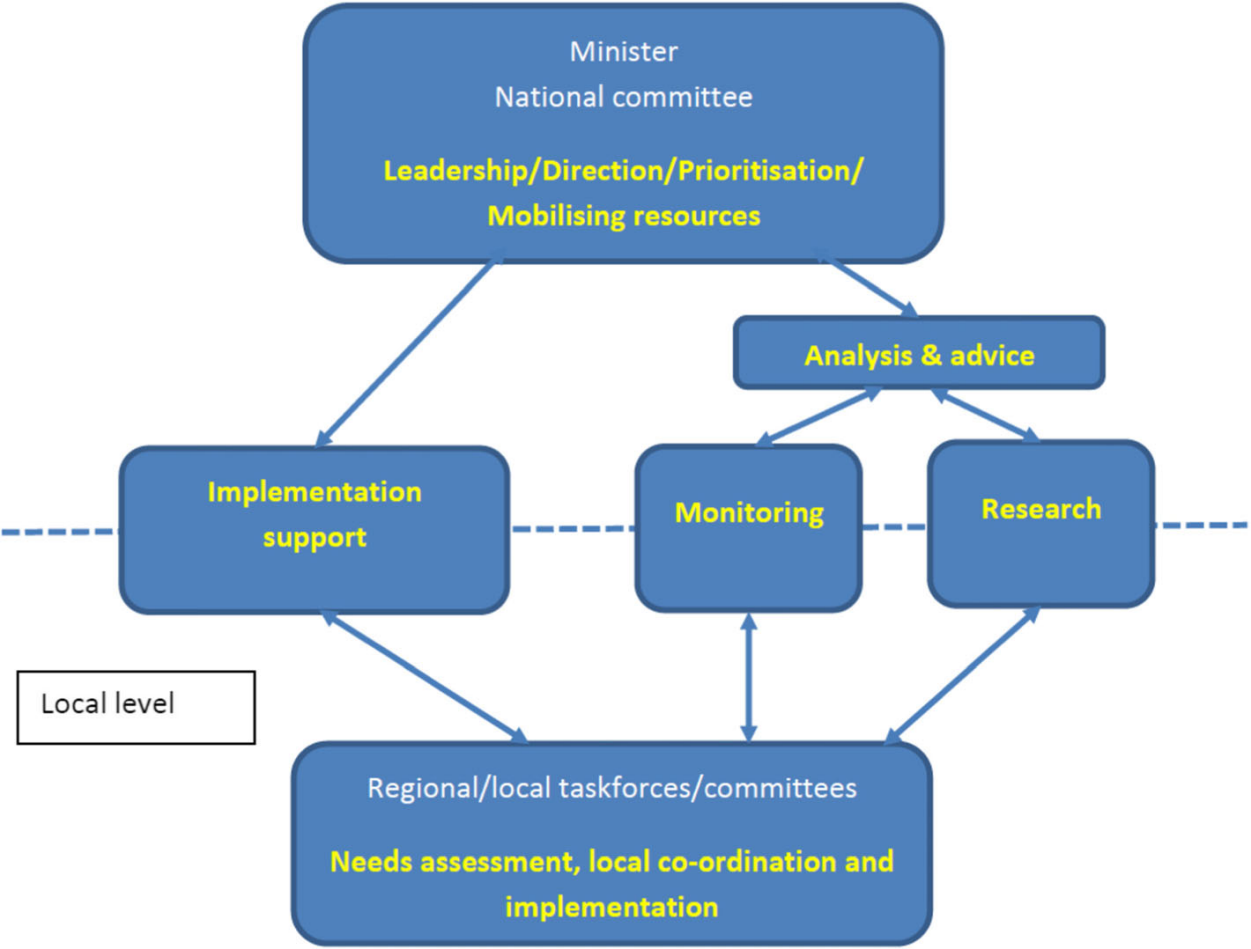
Proposed structure for the national drug strategy as depicted by the expert rapid review

An external expert review panel with invitees from the EMCDDA and elsewhere were asked to review existing drug strategy structures and consult with key structure personnel. Following this review, the structure in Fig. 1 was proposed.

Finally, a tender to conduct a review of evidence reviews was issued. Topics to be addressed were prevention, harm reduction and treatment and recovery. Reviews were based upon high-quality systematic reviews published since 2010. Authors reviewed 90 systematic reviews, which themselves included over 1700 primary research studies. Evidence was classified as category A: evidence suggests these intervention approaches may be effective; category B: evidence suggests these interventions approaches are not likely to be effective/may be harmful and category C: evidence is inconclusive or lacking on this intervention approach [1]. A summary of the evidence established is provided in Table 2.

**Table 2:** Evidence review of reviews results

May be effective	Unlikely to be effective	Inconclusive/lacking
Prevention		
Multi-component interventions set within multiple domains (e.g. school and community/family)	Brief interventions in school settings	Brief/motivational interventions in healthcare settings
Comprehensive school-based programmes (skills and social influence-based programmes)	School-based programmes focussing on increasing knowledge alone	Universal family-based interventions for drugs other than cannabis
Skills-development school based programmes	Mass-media campaigns alone (not as part of a multicomponent intervention)	Family-based interventions targeting high risk groups
Universal family interventions including parents and children for cannabis use		School-based programmes focussing on social influences alone
		Mentoring interventions
Harm reduction		
Needle and syringe programmes (enhanced by OST) in the community and prisons		Education in recreational/nightlife settings
Drug consumption rooms		Pill testing in recreational/nightlife settings
Overdose prevention with naloxone distribution		Staff training interventions in recreational/nightlife settings
Peer education training to prevent injection initiation		Targeted case finding for BBV testing
Multisession psychosocial/ educational interventions		Provision of dry blood spot BBV testing
Prison-based onsite HIV testing		Media campaigns
		Education for sex workers
		HIV risk reduction in prisons
Treatment and rehabilitation		
OST (methadone, buprenorphine) in community and prison settings	Pharmacological treatment for non-opiate drug use	Mindfulness-based treatments
Naltrexone implants	Cognitive behavioural therapy	Motivational interviewing
Multidimensional family therapy (young people’s cannabis use)	Acupuncture (alone)	Continuing care programmes
Couples-based therapy	Home visits and psychosocial interventions for pregnant women	Residential programmes (therapeutic community, 12-step)
Contingency management (short-term only benefits)		Physical activity interventions
CBT for people with PTSD		Boot camps for offenders
Peer-supported recovery, e.g. peer-coaching, recovery hosing, mutual aid		Drug diversion courts for offenders
Prison-based therapeutic communities		Psychosocial interventions for people with severe mental illness
		Pharmacological treatments for pregnant opiate users

### The partnership process, tensions, resolutions and lessons learned

In spite of the large committee size, there was no hierarchy in the seating positions. This enabled the Minister and the Chair to be seen and heard and to also to see each participating partner. Seating positions were not allocated in advance this enabled partners with common interests to sit together if they chose to and to create a common voice. This was often the case with the voluntary and community organisations, non-statutory partners and the family and service user organisations. Refreshments were available in the room before the meeting and this enabled informal exchanges to be made between partners and also alliances to be formed or opinions to be sought around an agenda item in advance of the formal meeting. While members of the committee were named individuals and were addressed in the meeting by their names, organisational name tags rather than personal names tags were used. This enabled partners to speak for their organisation and for the debate to remain non-personal. There were no microphones and meetings were not audio recorded. This provided some additional freedom of speech but also had the disadvantage of points raised possibly not being in the minutes either deliberately or inadvertently. The agenda was prepared in by the DPU and partnership input was not sought in advance but there was informal opportunity to raise non-agenda items at the meetings and these could be returned to at future meetings. This kept the agenda on track, it kept the strategy development progressing and the timing under the control of the DPU. It also prevented the agenda being hijacked by vested interests or stronger voices. Meetings often had relevant external guests providing a visual presentation detailing some aspect of the evidence. These external quests were hosted at the beginning of the meeting and were treated with additional respect, in a similar way a guest in one’s place of work might be treated. This set the tone for the meeting and this tone of mutual respect for the voice of each participant was maintained throughout the full development process and was a core component of the partnership.

Agreeing on the inclusion of alcohol and any related strategy as an item in the terms of reference was the first major challenge of the partnership. The item was presented at the first meeting in December 2015 and again each subsequent month in the second, third and fourth meeting. During this time, there was a national election and while the original party remained in power, there was a change in leadership and in government ministers. The alcohol debate, how it came to the committee several times, with members also proposing written suggestions and its final resolution after the Chair referred the matter back to the relevant new ministers, can be seen in the following excerpts from the monthly minutes:

The DPU informed the Committee that the Terms of Reference (ToR) had been revised based on discussions at the last meeting. … A number of members of the Committee, including the Community and Voluntary Sectors, the National Family Support Network (NFSN) and the Health Service Executive (HSE), expressed their reservations about the revised wording,… The Chair asked the Department to re-examine the wording (Item 3, Minutes, 12^th^ January 2016).

This contentious issue can be seen again in the next set of minutes:

The DPU informed the Committee that revised ToR had been circulated in advance of the meeting, with an alternate form of wording agreed by DPU and Tobacco and Policy Unit…The DPU representative noted that alternative proposals had been submitted by the Voluntary Sector and the HSE in advance of the meeting …The Community and Voluntary Sectors, the NFSN, the Regional Drug and Alcohol Task Force Chairs Network and the HSE, expressed their reservations about the revised wording on the basis of their understanding that previous Government … had envisaged an integrated policy on drugs and alcohol. The DPU representative said that the Department was not agreeable to re-naming the National Drugs Strategy…, the Chair informed the committee that he will meet with the Minister, when the new Government is appointed in order to get a clear direction on the terms of reference of the group. (Item 4. Minutes 9^th^ February 2016)

The matter arose again for the fourth time at the next meeting:

The Chair advised the Committee that the Terms of Reference will remain on the Agenda of the Committee, as the new Government has not yet been formed. His intention is to seek a direction from the Minister with responsibility for the Drugs Strategy, when appointed, on how alcohol is to be addressed within the new Strategy. (Item 5, Minutes 21^st^ March)

The lack of agreement on this aspect of the terms of reference can be clearly seen in the tone of the minutes and in the range of partners objecting, but it did not prevent the partnership making progress. The final resolution came in June 2016 in an email from the DPU 7 months after the inaugural meeting. It was interesting to note that while the Chair was waiting for a ministerial response, the terms of reference item was not on the agenda, nor was it on the agenda for the meeting in July 2016 following the ministerial decision and the decision of the ministers was accepted by the partners. The July meeting also had international external experts in attendance and partners respected both the visitors and the new ministers as there was still much work to be accomplished. However, partners were encouraged as the final wording of the item in the terms of reference did take into account concerns about linking strategies and the need to include alcohol. This can be seen in the final wording of the term of reference point 2 which stated:

Develop an integrated public health approach to substance misuse, which is defined as the harmful or hazardous use of psychoactive substances including alcohol and illicit drugs, incorporating the relevant recommendations of other related policies including the National Substance Misuse Strategy. ([7, 8], p10).

Additional challenges and lessons learned arose when non-statutory and statutory partners had opposing suggestions for actions. One example was the suggestion that as school buildings within disadvantaged communities were a possible community resource in terms of meeting rooms or space for youth activities, it was proposed that the Department of Education and Skills (DES) open these buildings for the community use after school. However, as this was a major change in operational procedures for the schools, this could not be agreed by the partner representative in attendance. A more senior representative was requested to attend the subsequent meeting by letter of invitation from the Chair. This finally resulted in Action 1.2.7 of the strategy which states that the DES will,

Facilitate increased use of school buildings, where feasible, for afterschool care and out of hours use to support local communities.’ ([7, 8], p28).

We can see within the wording of this action where a compromise position was adopted and accepted by both partners to enable the process to move forward.

A further challenge and a lesson learned during the evidence gathering stage of the process was to ensure that priorities identified in the epidemiological evidence, public consultation, evidence reviews, focus groups and expert review were not lost or omitted during the drafting stage of the policy development process. This is where the wide partnership role had its greatest impact and was of greatest benefit. As certain aspects of the background evidence were of a more pertinent interest to some groups than others, these groups could highlight, advocate for and keep track of their particular priority. Examples of this can be seen in the language of the policy and in the addiction nursing aspect of the policy. Throughout the drafting of the policy, the service user organisation representative could highlight any inadvertently stigmatising language. Similarly when the Irish Chair of the local chapter of the International Nurses Society on Addictions (IntNSA) was invited to participate in the focus group on the continuum of care, they could highlight the need for methadone prescribing by nurses to be explored within the new strategy and this recommendation was carried through from the focus group findings to the final draft of the strategy.

## Discussion

While not all members were fully satisfied with the strategy, the action plan was finalised and all partners welcomed the clear commitment to the health-led approach. Partnership was clearly articulated as one of the six values underpinning the strategy. The values were compassion, respect, equity, inclusion, partnership and evidence-informed. Actions within the strategy spanned five goals and these were promote and protect health and well-being; minimise the harms caused by the use and misuse of substances and promote rehabilitation and recovery; address the harms of drug markets and reduce access to drugs for harmful use; support participation of individuals, families and communities; and develop sound and comprehensive evidence-informed policies and actions. An implementation committee was established with many of the original committee members. This is chaired by the Minister and meets biannually to monitor progress on actions and to discuss barriers to implementation. A further operational subcommittee meets on a monthly basis to enable obstacles and barriers at the local service level to be addressed in a timely manner. Perhaps one important mistake in the development of these implementation committees was a lack of detail in the terms of reference, specifically with decisions on membership, succession planning, responsibility for and scope of agenda setting and monitoring of shorter term actions and outcomes from meetings. As indicated in the strategy action plan, a tender for the first medically supervised injecting facility was issued and a sub-committee to explore decriminalisation was formed.

## Conclusion

Using a genuine and committed partnership approach, where all voices at the table were heard, we succeeded in developing a sound strategy. While recognising that the partnership approach to policy development in Ireland is not novel, generalising a similar approach across jurisdictions could be initiated or piloted in the first instance. The independently chaired wide partnership approach with an agreed action plan based on trust, compromise and consensus to policy development is recommended. Success however depends on continuing support within the partnership and ongoing resourcing from the ministries. Early evidence of successful outcomes from the policy are not yet available, but annual monitoring reports of the action plan are scheduled and these will highlight barriers and enablers of progress and importantly the process of implementation.

## Data Availability

Not applicable

## References

[1] Bates G, Jones L, Maden M, Cochrane M, Pendlebury M, Sumnall H (2017). The effectiveness of interventions related to the use of illicit drugs: prevention, harm reduction, treatment and recovery. A ‘review of reviews.’.

[2] Citywide (2018). Stop the Stigma Campaign.

[3] Dean, G. (1984). Characteristics of heroin and non-heroin users in a North-Central Dublin area. *Medico-Social Research Board*.

[4] Dean G, Bradshaw J, Lavelle P (1983). Drug misuse in Ireland, 1982-1983. Investigation in a north central Dublin area and in Galway, Sligo and Cork.

[5] Dean G, Kelly G (1985). The opiate epidemic in Dublin.

[6] Department of Community, Rural and Gaeltacht Affairs (2009). National Drug Strategy (interim) 2009-2016.

[7] Department of Health (2017). Reducing Harm, Supporting Recovery- A health led response to drug and alcohol use in Ireland 2017-2025.

[8] Department of Health (2017). Report on Public Consultation undertaken to inform the new National Drugs Strategy.

[9] Department of Health. (2018). Drugs Policy [Government]. Retrieved April 5, 2018, from http://health.gov.ie/healthy-ireland/drugs-policy/

[10] Department of The Taoiseach (2000) Partnership 2000 for inclusion and competitiveness. Government of Ireland, Dublin. Retrieved from www.ictu.ie/download/pdf/partnership_2000.pdf. 21 Aug 2019.

[11] Department of Tourism, Sport and Recreation (2001). Building on experience: National Drugs Strategy 2001-2008.

[12] EMCDDA (2017). New developments in national drug strategies in Europe.

[13] Giese C, Igoe D, Gibbons Z, Hurley C, Stokes S, McNamara S, et al. Injection of new psychoactive substance snow blow associated with recently acquired HIV infections among homeless people who inject drugs in Dublin, Ireland, 2015. *Eurosurveillance*. 2015;*20*(40). 10.2807/1560-7917.ES.2015.20.40.30036 26537764

[14] Giraudon I, Matias J, Vicente J (2013). Key epidemiological indicator drug-related deaths and mortality among drug users. EMCDDA.

[15] National Advisory Committee on Drugs (2016). Estimating the prevalence of opiate use in Ireland using indirect statistical methods.

[16] National Advisory Committee on Drugs and Alcohol, & Department of Health, Social Services and Public Safety. (2016). Prevalence of drug use and gambling in Ireland & drug use in Northern Ireland. Bulletin 1. National Advisory Committee on Drugs and Alcohol. Retrieved from https://www.drugsandalcohol.ie/26364/

[17] Kelly A, Teljeur C, Carvalho M, National Advisory Committee on Drugs (2009). Prevalence of opiate use in Ireland 2006: a 3-source capture recapture study.

